# Supplementary material for: A robot for high yield electrophysiology and morphology of single neurons in vivo
Source: Nat Commun. 2017 Jun 1;8:15604. doi: 10.1038/ncomms15604 (PMC5461495; doi:10.1038/ncomms15604)
Supplement: Supplementary Information — Supplementary Figures [file ncomms15604-s1.pdf]

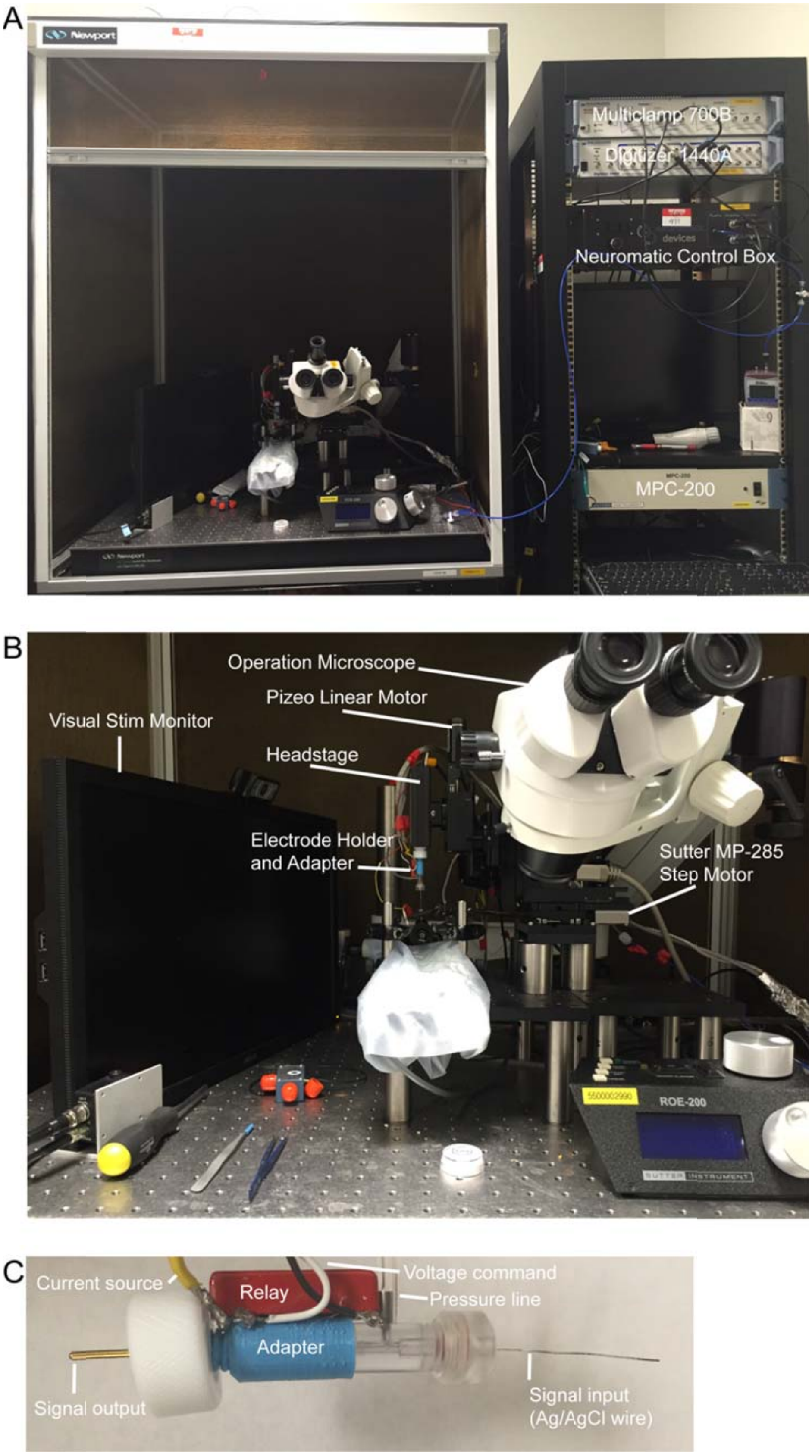

A
Multiclamp 700B
Digitizer 1440A
Neuromatic Control Box
MPC-200
B
Operation Microscope
Pizeo Linear Motor
Visual Stim Monitor
Headstage
Electrode Holder
and Adapter
Sutter MP-285
Step Motor
ROE-200
C
Current source
Relay
Voltage command
Pressure line
Adapter
Signal output
Signal input
(Ag/AgCl wire)

Supplementary Figure 1. ACE robotic platform. **A.** Overview of the rig setup showing major hardware components of ACE (Automatic single-Cell Experimenter) including the MultiClamp 700B, Digidata 1440A, Neuromatic control box and Sutter MP-285 control unit MPC-200. The Axoporator800A (current source for electroporation) is not shown in this picture. **B.** Experiment setup for recording and labeling single neurons in mouse primary visual cortex (V1). Electrode holder adapter assembly is installed on the MultiClamp 700B headstage attached to a piezo linear drive, which is mounted onto a Sutter MP-285 4-axis micromanipulator. **C.** Close-up view of the electrode holder-adapter-switching relay assembly.

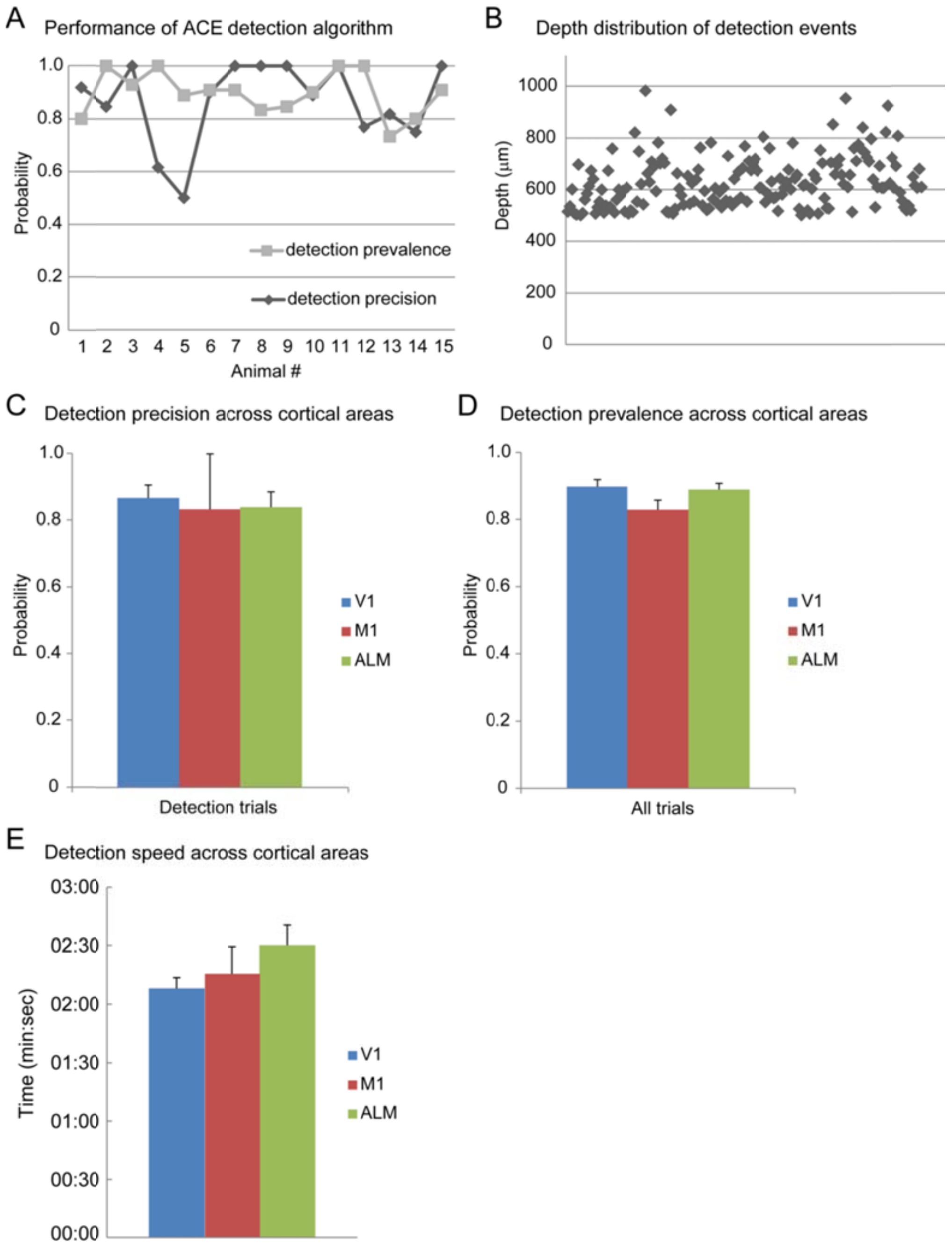

Supplementary Figure 2. ACE reliably detects neurons in the brain with high efficiency. **A.** High detection prevalence (gray line) and precision (black line) of ACE in mouse V1 cortices (n = 15). ACE achieved stable performance in detecting single neurons in vivo across 15 animals. **B.** Distribution of the subpial depth of detection events as in **A** (168 penetrations in 15 mice). **C-E**. Independence of ACE performance on brain regions. The Detection precision (**C**), Detection prevalence (**D**) and Detection time (**E**) are comparable between sensory (V1), motor (M1) and frontal (ALM) cortical areas.

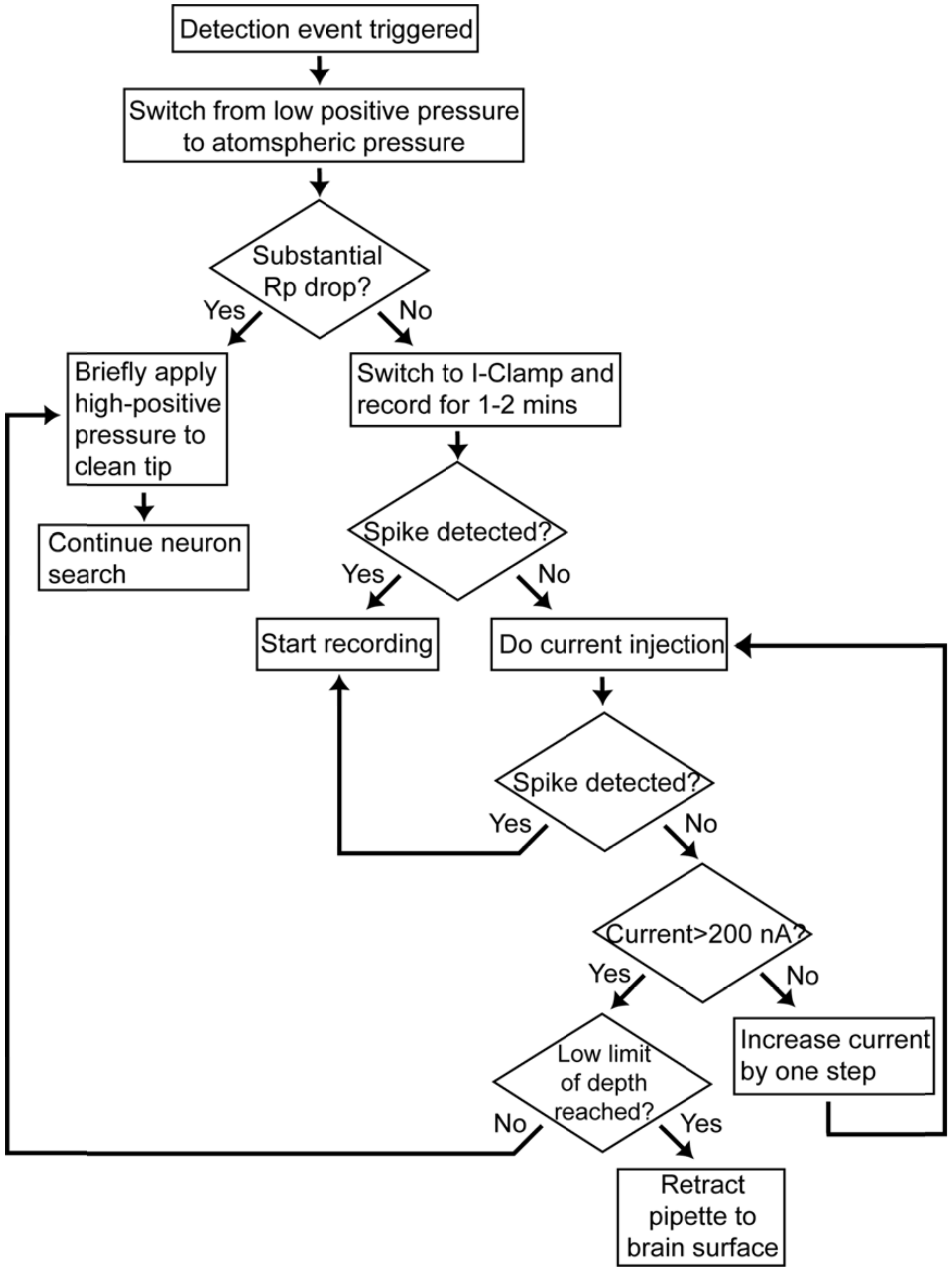

Supplementary Figure 3. Decision tree for current injection in ACE.

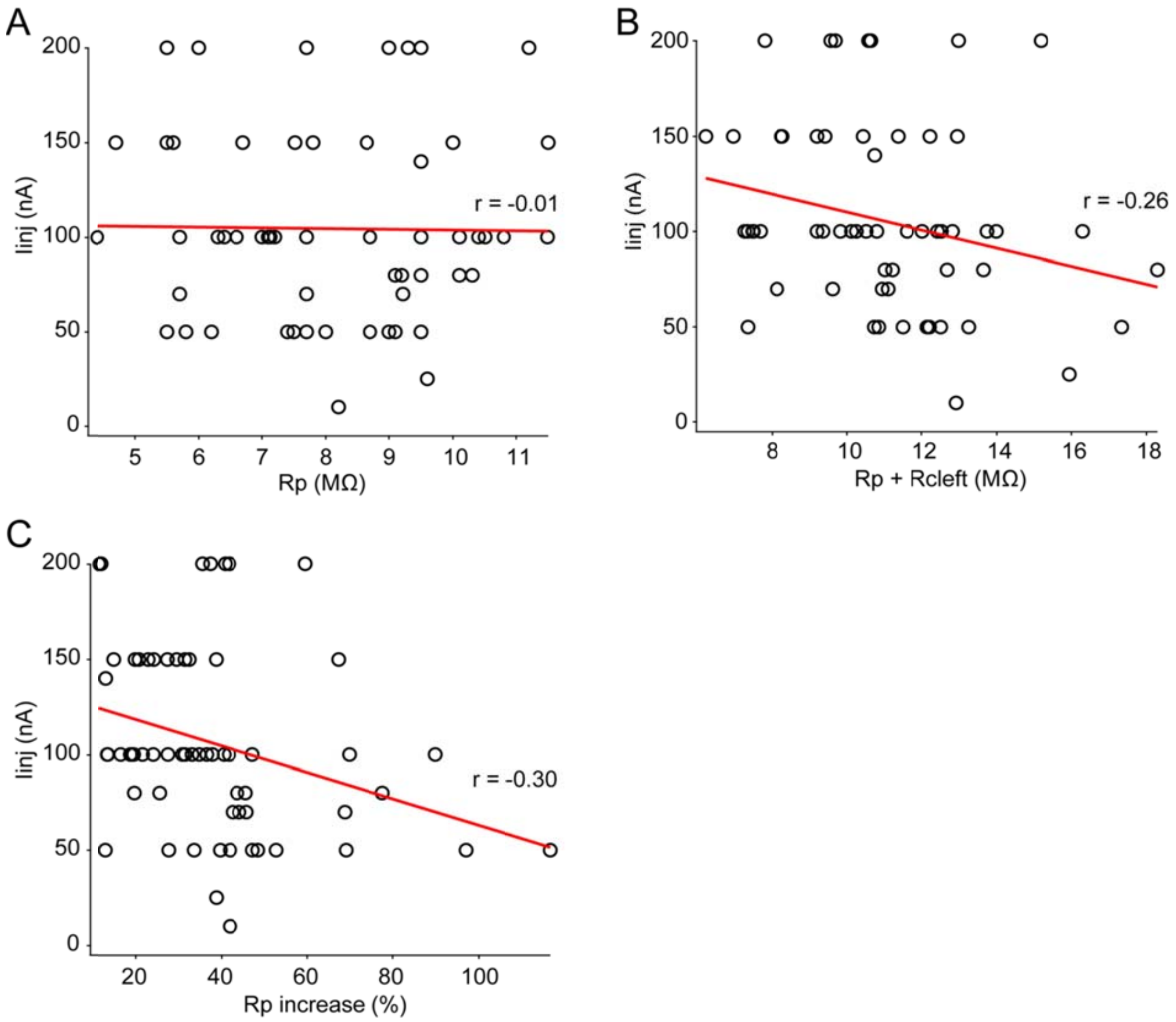

Supplementary Figure 4. The amplitude of current injected to evoke spikes after detection is not correlated with the pipette resistance (Rp) or seal resistance (Rp + Rcleft). **A.** The amplitude of current injected (Iinj) measured in manual trials (n = 61) is not dependent on Rp (r = -0.01). **B.** Iinj measured in manual trials is weakly correlated with the seal resistance Rp + Rcleft (r = -0.26). Rp + Rcleft was measured at the atmospheric pressure, after the low positive pressure was released. **C.** Iinj in manual trials is weakly correlated with the relative Rp increase (Rcleft/Rp X 100%, r = -0.30).

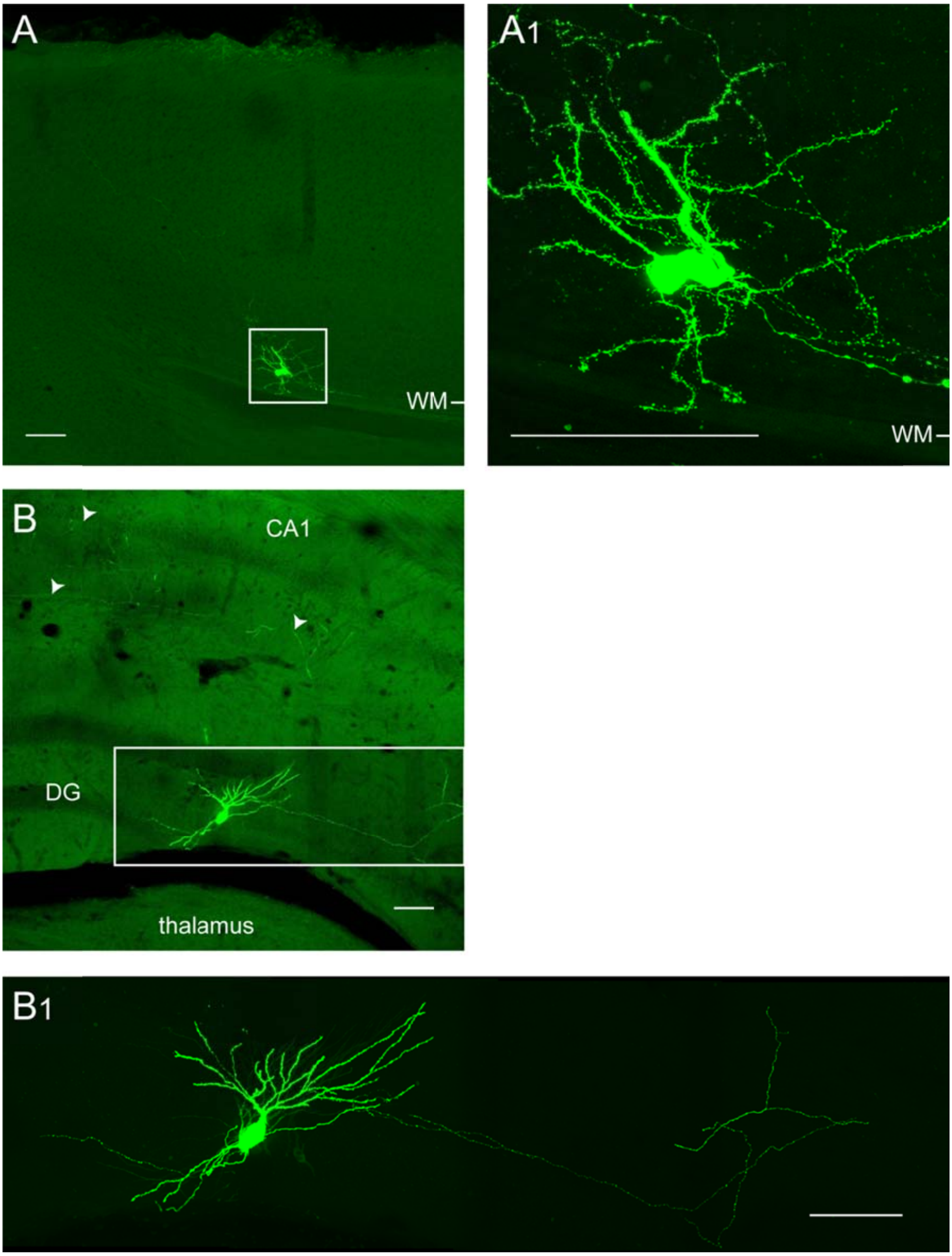

A
WM
A1
WM
B
CA1
DG
thalamus
B1

Supplementary Figure 5. ACE successfully labels neurons in deep brain structures. **A.** Z-projection image of a confocal image stack showing the overview of two neighboring L6 neurons electroporated by ACE with EGFP plasmid in V1 *in vivo*. High electroporation voltage (-20V) was used and the animal was allowed to survive for 7 days before perfusion. Native fluorescence was imaged without any antibody amplification with a 10X, 0.4 NA dry objective. **A1**. Z-projection image showing the close-up view of the soma-containing region of these 2 L6 neurons. Confocal stack was taken with a 63X, 1.4 NA oil-immersion objective. WM: white matter. **B.** Montage of Z-projection images of confocal image stacks of a labeled hippocampal neuron electroporated by ACE with EGFP plasmid *in vivo*. Image stacks of native fluorescence were taken with a 10X, 0.4 NA dry objective. Arrow heads: projecting axons from CA3 to CA1. The soma region within the white box is shown in **B1**. **B1**: Montage of Z-projection images of confocal image stacks (with a 40X, 1.3 NA oil-immersion objective). Note the fine detail of dendritic and axonal structure, which confirms the high labeling quality. Expression time is 7-day in this animal and native fluorescence was imaged. DG: dentate gyrus. Scale bar: 100 μm.

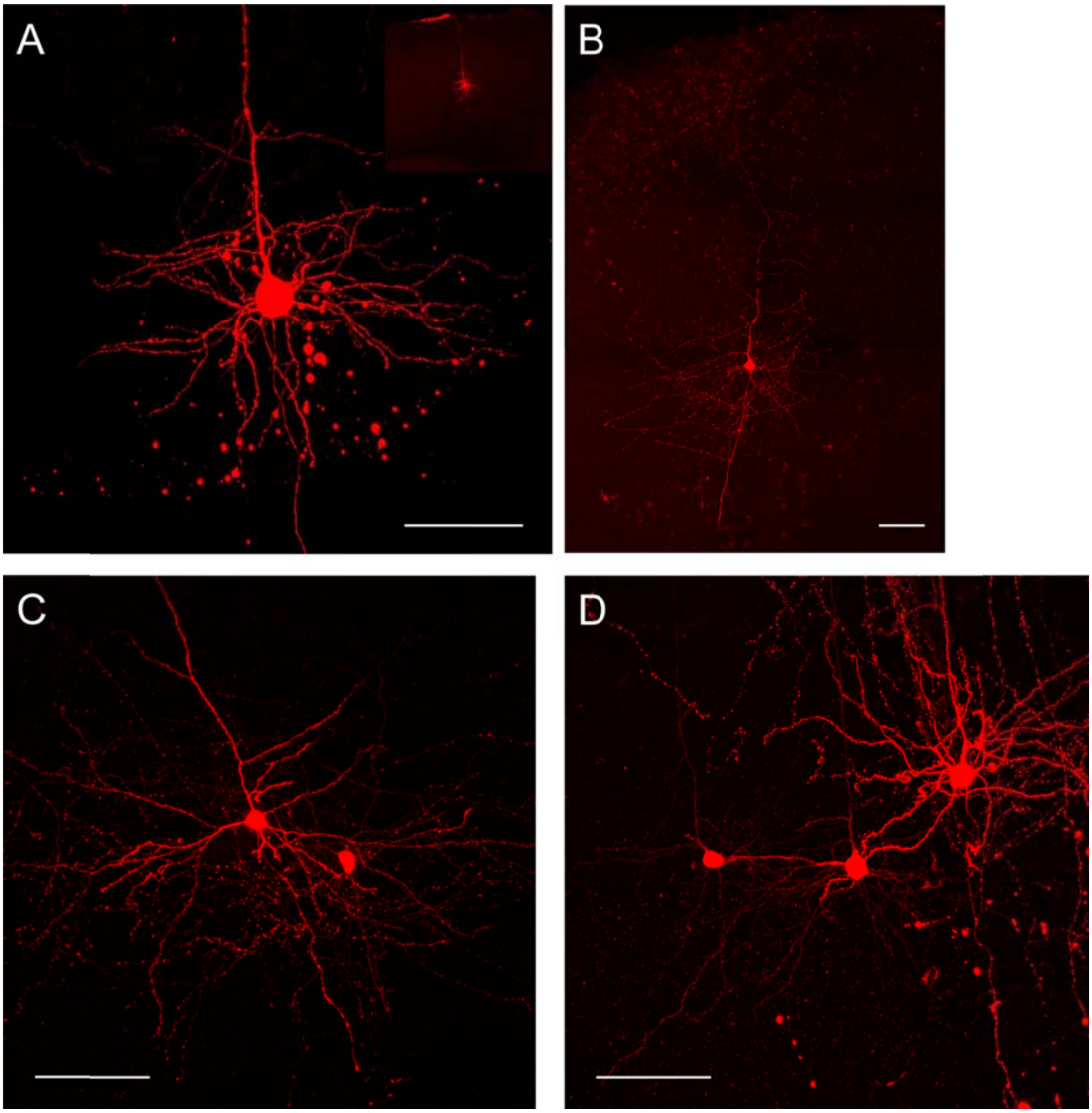

Supplementary Figure 6. ACE works with multiple plasmids. **A** and **B**. ACE labels single neurons with CAG-TdTomato plasmid in mouse V1. **C** and **D**. Multiple neighboring neurons (both excitatory and inhibitory) labeled by ACE with high electroporation voltages (-20V in **C** and -40 V in **D**). Montages of Z-projection images of confocal image stacks are shown here. Native fluorescence was imaged with a 40X, 1.3 NA oil-immersion objective. Scale bar: 100 μm.

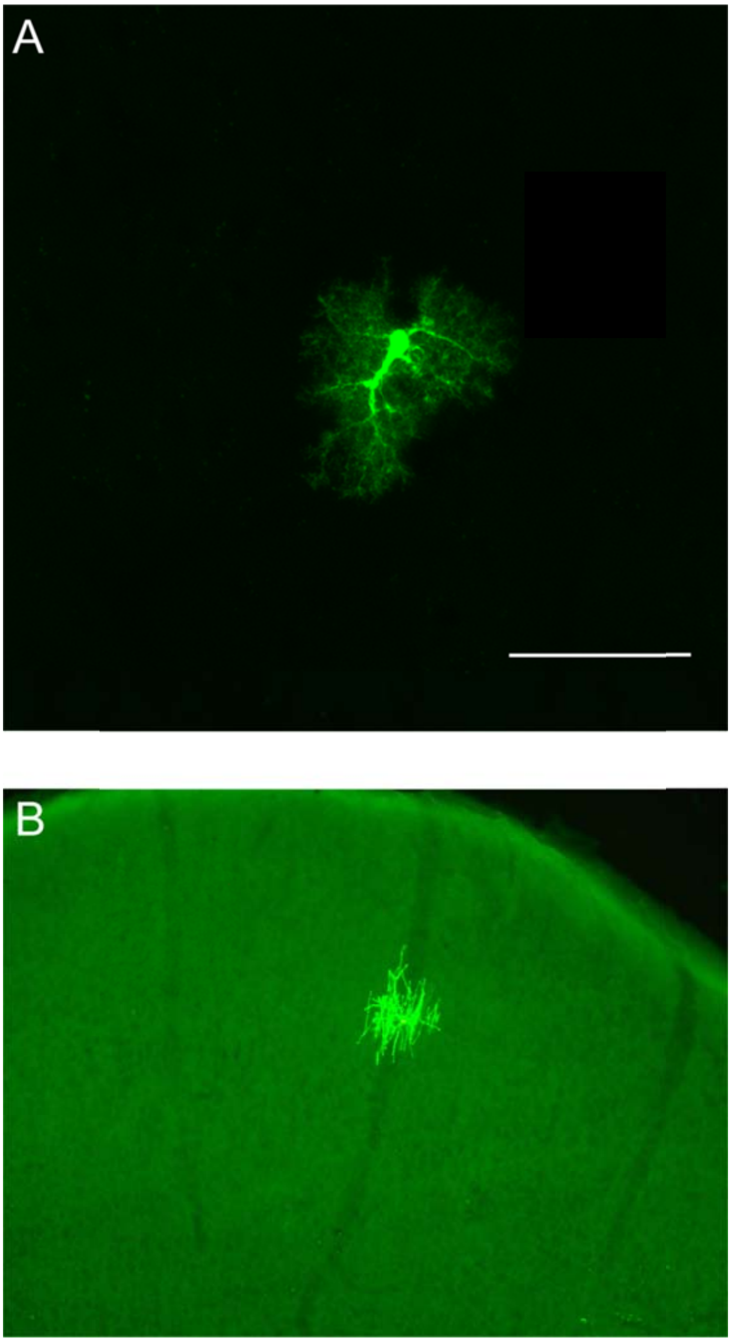

Supplementary Figure 7. ACE labels single glial cells in the brain. A. Example single Astrocyte recovered in mouse V1, expressing EGFP after elelctroporation. B. Example single Oligodendrocyte in mouse V1 expressing EGFP after elelctroporation. Scale bar: 100 μm.

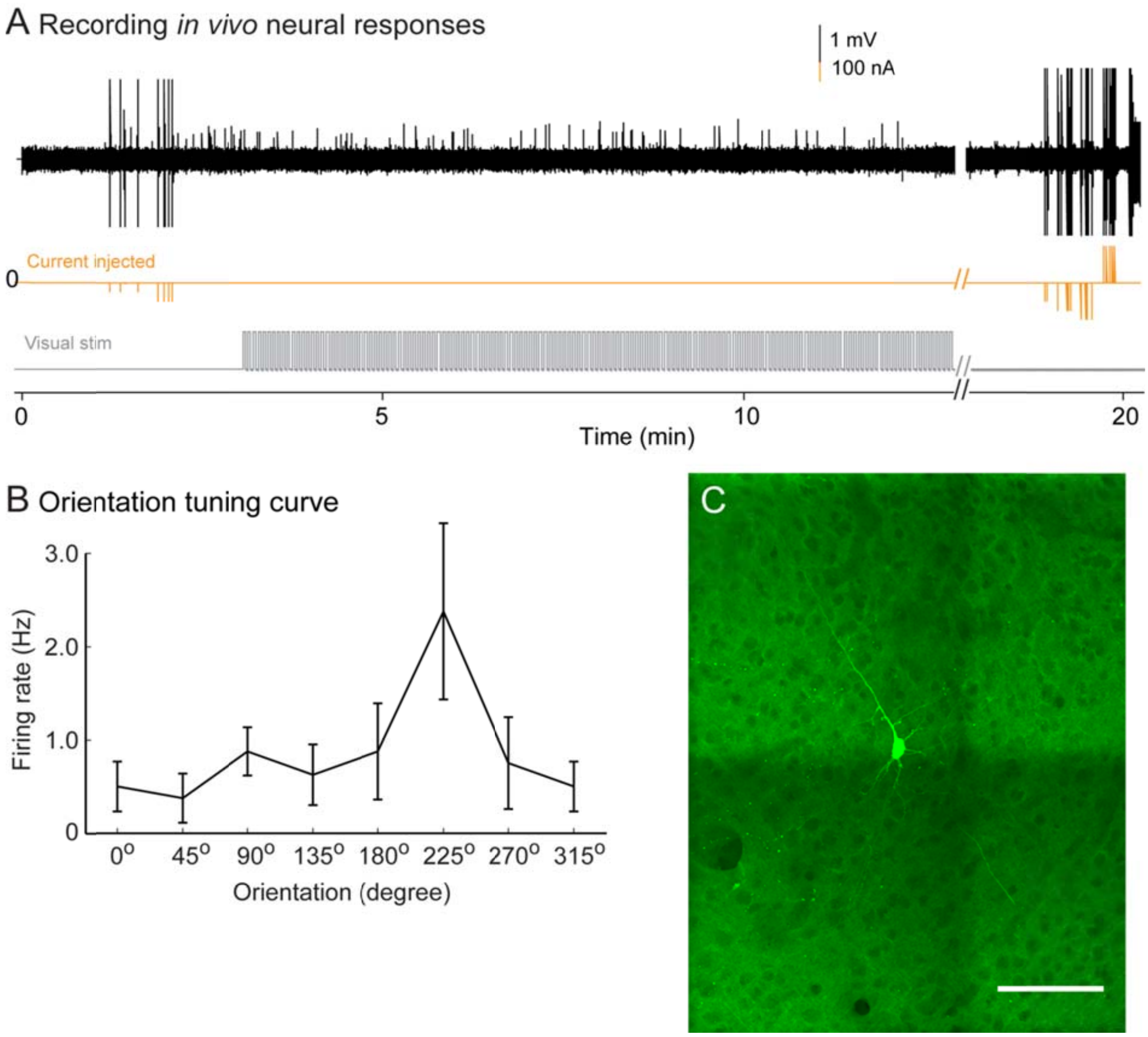

Supplementary Figure 8. ACE recovers neuronal morphology after electrophysiological characterization. **A**. Electrophysiological recording of a neuron detected by ACE at 598 μm underneath the pia. Responses to drifting gratings (8 orientations, 3 spatial frequencies and 1 temporal frequency, 8 repetitions) were recorded. Note that activity was sparse in this neuron. **B**. Orientation tuning curve of neuron in **A** to its preferred spatial frequency (0.02 cycle per degree). Error bars represent SEM. **C.** Z-projection image of confocal image stacks showing the morphology of the recorded neuron after 4-day expression of EGFP. Native EGFP fluorescence was imaged with a 40X, 1.3 NA oil-immersion objective. Scale bar: 100 μm.

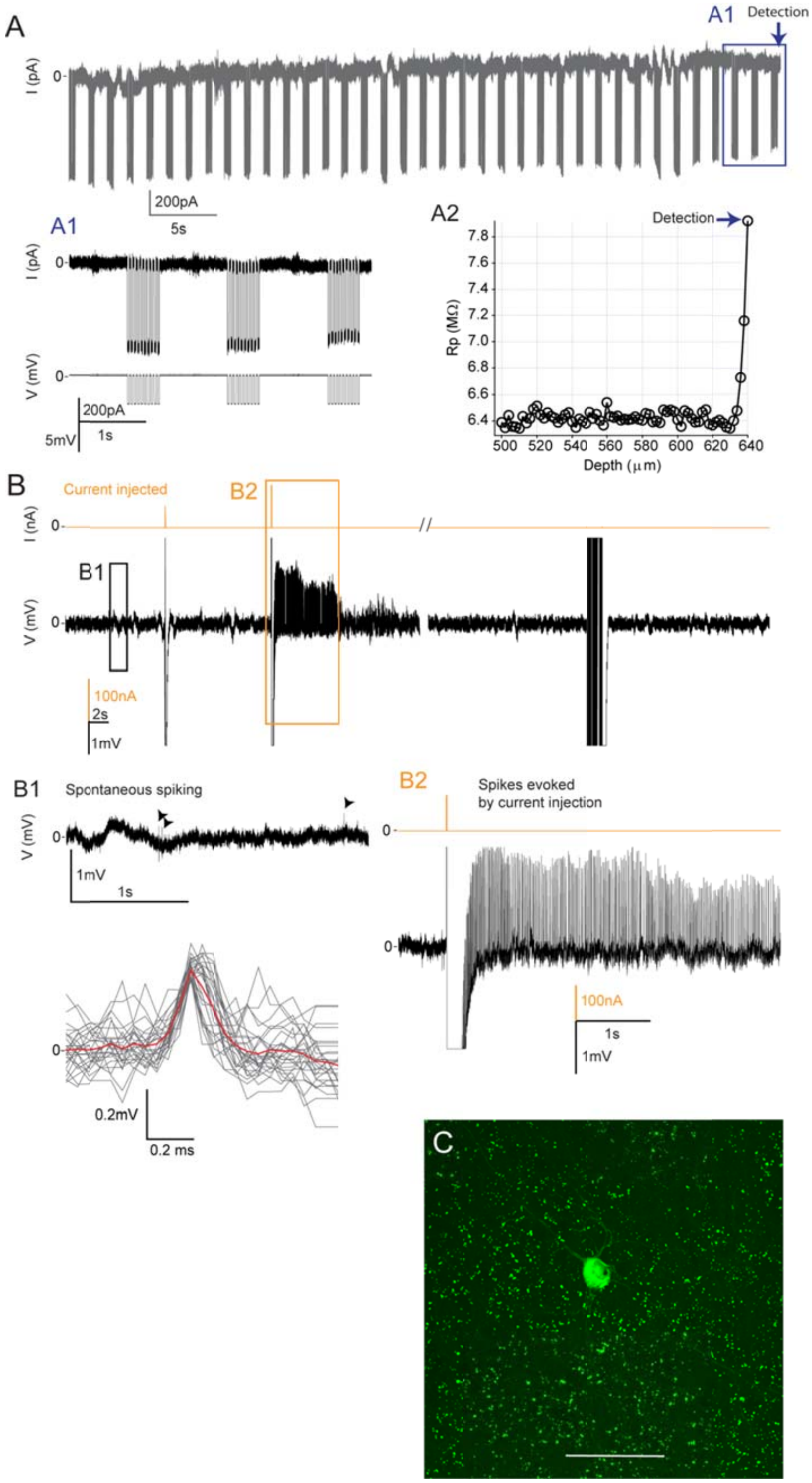

A
A1
Detection
I (pA)
0
200pA
5s
A1
I (pA)
0
V (mV)
0
200pA
5mV
1s
A2
Detection
Rp (MΩ)
7.8
7.6
7.4
7.2
7.0
6.8
6.6
6.4
500 520 540 560 580 600 620 640
Depth (μm)
B
Current injected
B2
I (nA)
0
B1
V (mV)
0
100nA
2s
1mV
B1
Spontaneous spiking
V (mV)
0
1mV
1s
0
0.2mV
0.2 ms
B2
Spikes evoked
by current injection
0
0
100nA
1s
1mV
C

Supplementary Figure 9. ACE successfully records and labels single fast-spiking (FS) aspiny neurons *in vivo*. **A.** Recorded current trace showing ACE neuron detection of one FS neuron. The pipette was advanced into the brain at 2 μm/step/sec. **A1**. At each step, a train of 10 -5 mV test pulses were delivered to the pipette and the current responses were measured to calculate the average pipette resistance (Rp). Rp was then compared with values calculated at previous steps to see whether the neuron detection criteria would be met. **A2.** Plot of Rp vs. depth of the entire penetration. Detection occurred within 4-6 μm at 640mm. A threshould of 10% Rp increase was used. **B.** Recording of neural activity in the detected FS neuron and electroporation. **B1.** Recorded voltage traces showing spontaneous spikes (arrow heads) under current clamp (top). Bottom: Average spike waveform (red) from 27 spontaneously fired spikes (gray). Note the narrow spike width (half-height half-width of 0.16 ± 0.01 ms), which is typical for fast-spiking neurons. **B2.** Mild current injection (100 nA) evoked high frequency (~200 Hz) and reversible spiking. All these features suggest a fast-spiking inhibitory interneuron. **C.** Z-projection image of a confocal image stack showing the smooth morphology of the same neuron recorded and electroporated by ACE with EGFP plasmid *in vivo*. Native fluorescence was imaged with a 63X, 1.4 NA oil-immersion objective. Expression time is 4-day. Compared with labeled pyramidal neurons in the same animal, labeling in 3 FS neurons is consistently weak, suggesting a cell-type dependence of GFP expression. Scale bar: 51 μm.

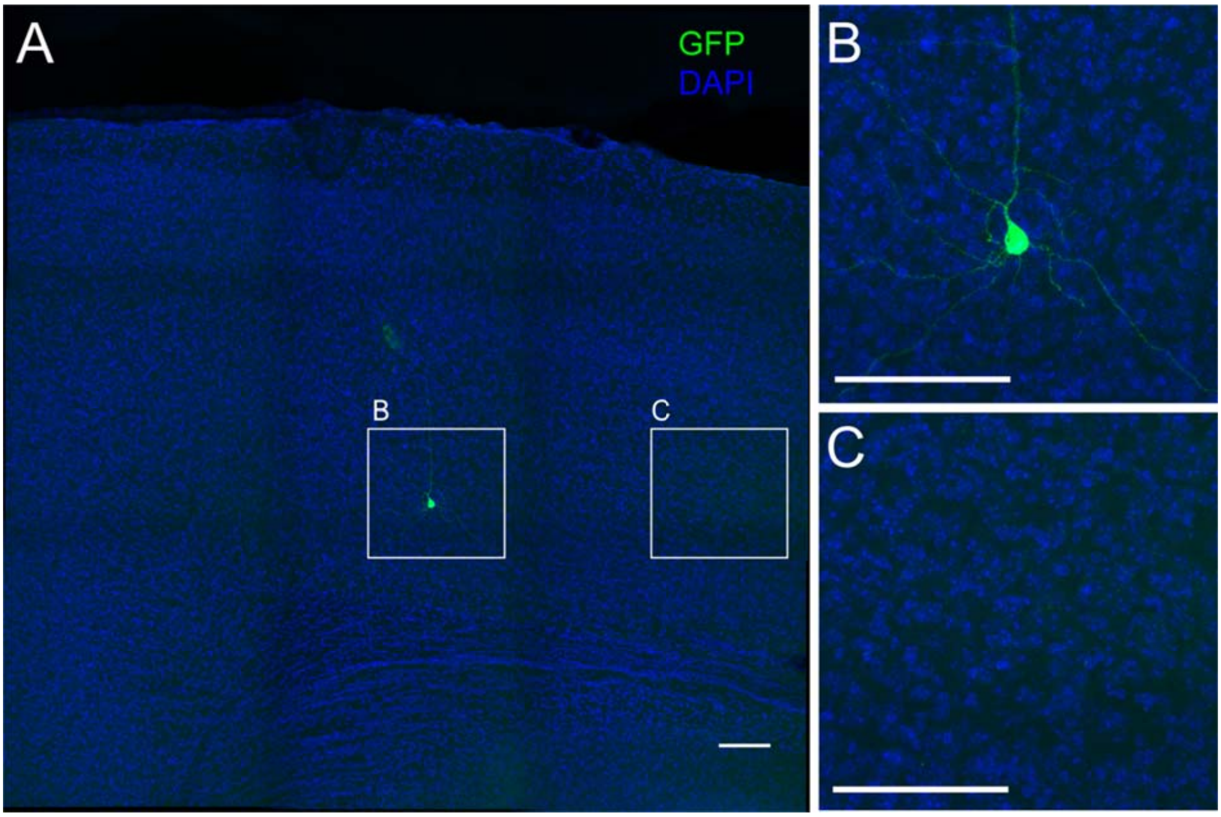

Supplementary Figure 10. ACE has little/no negative influence on the healthiness of brain tissue after recording and eletcroporation. **A**. Montage of Z-projection images of confocal image stacks of a labeled V1 L5 neuron electroporated by ACE with EGFP plasmid *in vivo*. DAPI staining was conducted before image stacks were taken with a 10X, 0.4 NA dry objective. Note the cleanness of the labeling. Expression time is 4-day. **B** and **C**. Close-up view of the soma-containing region (**B**) and a size-comparable adjacent region (**C**) within the same coronal section. No apparent difference in cell density is noticed. Scale bar: 100 μm.
